# Machine Learning Prediction of Prediabetes in a Young Male Chinese Cohort with 5.8-Year Follow-Up

**DOI:** 10.3390/diagnostics14100979

**Published:** 2024-05-08

**Authors:** Chi-Hao Liu, Chun-Feng Chang, I-Chien Chen, Fan-Min Lin, Shiow-Jyu Tzou, Chung-Bao Hsieh, Ta-Wei Chu, Dee Pei

**Affiliations:** 1 Division of Nephrology, Department of Internal Medicine, Kaohsiung Armed Forces General Hospital, Kaohsiung 802, Taiwan; colinliu1201@gmail.com; 2 Divisions of Urology, Department of Surgery, Kaohsiung Armed Forces General Hospital, Kaohsiung 802, Taiwan; ccf701221@gmail.com; 3 Divisions of Urology, Department of Surgery, Tri-Service General Hospital, National Defense Medical Center, Taipei 114, Taiwan; 4 Department of Nursing, Kaohsiung Armed Forces General Hospital, Kaohsiung 802, Taiwan; sammi5493@gmail.com; 5 Division of Pulmonary Medicine, Department of Internal Medicine, Kaohsiung Armed Forces General Hospital, Kaohsiung 802, Taiwan; glutamate31@hotmail.com; 6 Teaching and Researching Center, Kaohsiung Armed Forces General Hospital, Kaohsiung 802, Taiwan; jyu0120@gmail.com; 7 Institute of Medical Science and Technology, National Sun Yat-sen University, Kaohsiung 804, Taiwan; 8 Department of Surgery, Kaohsiung Armed Forces General Hospital, Kaohsiung 802, Taiwan; albert0920@yahoo.com.tw; 9 Department of Obstetrics and Gynecology, Tri-Service General Hospital, National Defense Medical Center, Taipei 114, Taiwan; taweichu@gmail.com; 10 MJ Health Research Foundation, Taipei 114, Taiwan; 11 Division of Endocrinology and Metabolism, Department of Internal Medicine, Fu Jen Catholic University Hospital, School of Medicine, College of Medicine, Fu Jen Catholic University, New Taipei 243, Taiwan

**Keywords:** machine learning, prediabetes, young men, Chinese

## Abstract

The identification of risk factors for future prediabetes in young men remains largely unexamined. This study enrolled 6247 young ethnic Chinese men with normal fasting plasma glucose at the baseline (FPG_base_), and used machine learning (Mach-L) methods to predict prediabetes after 5.8 years. The study seeks to achieve the following: 1. Evaluate whether Mach-L outperformed traditional multiple linear regression (MLR). 2. Identify the most important risk factors. The baseline data included demographic, biochemistry, and lifestyle information. Two models were built, where Model 1 included all variables and Model 2 excluded FPG_base,_ since it had the most profound effect on prediction. Random forest, stochastic gradient boosting, eXtreme gradient boosting, and elastic net were used, and the model performance was compared using different error metrics. All the Mach-L errors were smaller than those for MLR, thus Mach-L provided the most accurate results. In descending order of importance, the key factors for Model 1 were FPG_base_, body fat (BF), creatinine (Cr), thyroid stimulating hormone (TSH), WBC, and age, while those for Model 2 were BF, white blood cell, age, TSH, TG, and LDL-C. We concluded that FPG_base_ was the most important factor to predict future prediabetes. However, after removing FPG_base_, WBC, TSH, BF, HDL-C, and age were the key factors after 5.8 years.

## 1. Introduction

Globally, type 2 diabetes (T2D) is the most common type of diabetes, and its prevalence has increased drastically in recent years. In 2022, according to the American Diabetes Association, over 11% of Americans are diabetic, with type 2 accounting for 95% of all cases [1]. The prevalence and ratios of type 1 and type 2 diabetes in Taiwan are similar. According to Taiwan Biobank, in 2020, Taiwan had 2.18 million diabetic patients (11.1% of the population). Again, type 1 diabetes only accounted for 0.51% of these patients [2]. From 2001 to 2017, the number of T2D cases among subjects younger than 20 years old nearly doubled [3], while the number of cases in subjects under the age of 35 increased 2.8-fold [4]. These reports indicate that the age of initial diabetes onset has been decreasing. Since the severity of diabetes complications is related to the time of onset, patients who develop diabetes at a younger age will suffer more extensive and severe complications [5]. This raises an urgent need for early diagnosis and management among younger people susceptible to T2D.

Many risk factors have been identified for susceptibility to diabetes, including being overweight, smoking, alcohol consumption, income, less physical activity, marital status, and educational level [6]. Most previous studies of diabetes susceptibility relied on traditional statistic methods such as multiple linear regression (MLR). In recent years, machine learning (Mach-L) techniques have been widely applied in many fields including medicine [7,8]. Mach-L applies computer algorithms to achieve our goal automatically on the basis of Mitchell [9]. Mach-L can capture nonlinear relationships in the data and complex interactions among multiple predictors, allowing it to potentially outperform other conventional multiple logistic regression for diseases [10]. Several large-cohort studies have focused on the prediction of prediabetes, but have failed to account for factors including lifestyle, income, education level, and marriage status. The present study enrolls subjects under the age of 36, with a follow-up of 5.8 years. Four different Mach-L methods are applied to achieve the following:

1. Compare Mach-L and MLR performance in predicting future prediabetes
2. Identify and rank the six most important risk factors for prediabetes.

## 2. Materials and Methods

### 2.1. Subject Selection

**Figure 1:**
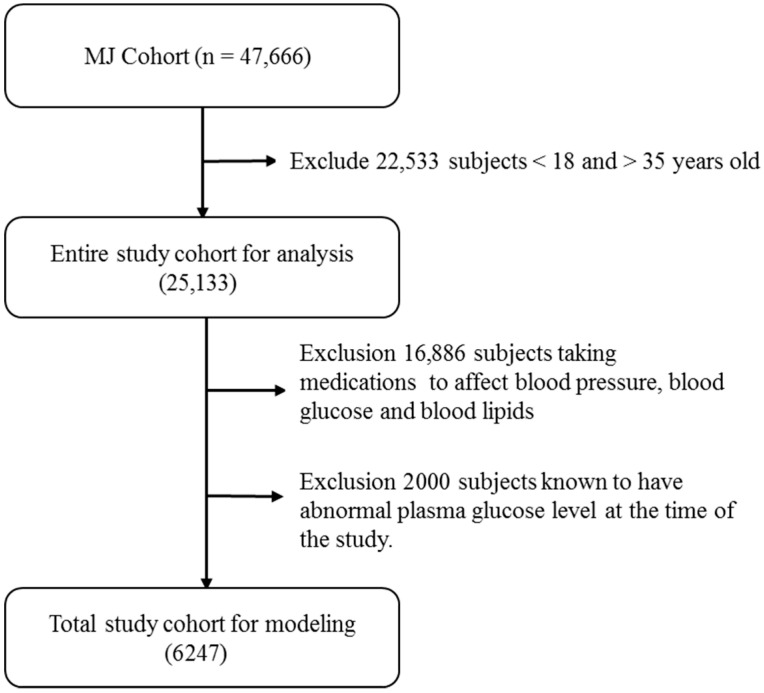
Participant selection.

The data for this study were sourced from the Taiwan MJ Cohort, an ongoing prospective cohort of health examinations conducted by the MJ Health Screening Centers in Taiwan [11]. These examinations cover more than 100 important biological indicators, including anthropometric measurements, blood tests, imaging tests, etc. Each participant completed a self-administered questionnaire, covering personal and family medical history, current health status, lifestyle, physical exercise, sleep habits, and dietary habits [12]. All participants provided informed consent. All or part of the data used in this research were authorized by and received from the MJ Health Research Foundation (Authorization Code: MJHRF2023007A). Any interpretations or conclusions described in this paper do not represent the views of MJ Health Research [13]. The study protocol was approved by the Institutional Review Board of the Kaohsiung Armed Forces General Hospital (IRB No.: KAFGHIRB 112-006). An initial sample of 23,462 subjects under the age of 36 was selected based on the standards of care published by the American Diabetes Association [14], which notes that most T2D diagnoses occur after this age. Excluding subjects who did not fit our inclusion criteria left a total sample of 6247 male subjects for further analysis (Figure 1).

The exclusion criteria were as follows:

1. Age < 18 and >35 years old;
2. Taking any medications known to affect blood pressure, blood glucose, or blood lipids;
3. Abnormal plasma glucose level at the time of the study.

The following methods were published in our previous study [15]. On the day of the study, senior nursing staff recorded the subject’s medical history, including current medications, and a physical examination was performed. Body fat percentage (BF) was measured using bioelectrical impedance analysis. WBC, hemoglobin levels, and the platelet count (Plt) were measured using standard laboratory techniques, typically performed on automated hematology analyzers. Creatinine (Cr), uric acid (UA), and C-reactive protein (CRP) levels were measured through blood tests using a biomedical analyzer to assess the concentration of these substances in the blood [16].

Following previously published protocols, demographic and biochemical data were collected as follows. After fasting for 10 h, blood samples were collected for biochemical analysis. Plasma was separated from blood within 1 h of collection and stored at 30 °C until the analysis of the fasting plasma glucose and lipid profiles. The FPG was measured using the glucose oxidase method (YSI 203 glucose analyzer; Yellow Springs Instruments, Yellow Springs, OH, USA). The total cholesterol and triglyceride (TG) levels were measured using the dry multilayer analytical slide method with a Fuji Dri-Chem 3000 analyzer (Fuji Photo Film, Tokyo, Japan). The serum high-density lipoprotein cholesterol and low-density lipoprotein cholesterol concentrations were analyzed using an enzymatic cholesterol assay, following dextran sulfate precipitation. A Beckman Coulter AU 5800 biochemical analyzer was used to determine the urine ACR via turbidimetry (Indianapolis, IN, USA).

Table 1 shows the 25 baseline variables, including the participants’ age, body fat, complete blood cell count, biochemistries, thyroid stimulating hormone, C-reactive protein, education level, marital status, and income level. Alcohol consumption was defined as the multiple of the total consumption duration, frequency, and alcohol percentage. Similarly, smoking was the multiple of the smoking duration, frequency, and number of cigarettes. The sport area was the multiple of the exercise duration, frequency, and type. All of these parameters were used as independent variables, while the dependent variable was the fasting plasma glucose (FPG_end_) after a 5.8-year follow-up, on average.

### 2.2. Traditional Statistics

Two models were built in the present study. From our preliminary evaluation, Model 1 included all 25 variables. Our results showed that the FPG_base_ displayed 100% importance when compared to the second important factor (BF, 28.3%). To further evaluate the hidden interactions between these factors, Model 2 was built without the baseline FPG.

Data are represented as means ± standard deviations. The Student’s t test was used to evaluate the differences in the continuous data between married and unmarried participants. Education and income levels were used as ordinal variables for analysis of variance (ANOVA). Pearson’s correlation was used to analyze the relationships between all the continuous risk factors and the FPG_end_ (Table 2). All statistical tests were two sided, and *p* < 0.05 was considered statistically significant. Statistical analysis was performed using SPSS 10.0 for Windows (SPSS, Chicago, IL, USA).

### 2.3. Proposed Machine Learning Scheme

Building on our group’s previous work, models were constructed using four different Mach-L methods to predict prediabetes and to rank risk factors [15].

Random forest (RF) is an ensemble learning decision tree algorithm that combines bootstrap resampling and bagging [17]. RF’s randomly generates many different and unpruned CART decision trees, using the decrease in Gini impurity as the splitting criterion. The trees in the forest are then averaged or voted on to generate output probabilities and a final model, producing a robust model [18]. The following methods were published by our group [15,19]:

Stochastic gradient boosting (SGB) is a tree-based gradient boosting learning algorithm that combines bagging and boosting techniques to minimize the loss function and solve the overfitting problem of traditional decision trees [20]. In SGB, many stochastic weak learners of trees are sequentially generated through multiple iterations, in which each tree concentrates on correcting or explaining errors of the tree generated in the previous iteration. That is, the residual of the previous tree iteration is used as the input for the newly generated tree. This iterative process is repeated until the convergence condition, or a stopping criterion is reached for the maximum number of iterations. Finally, the cumulative results of many trees are used to produce a robust model.

The third method used in this study is eXtreme gradient boosting (XGBoost), a gradient boosting technique based on an optimized extension of SGB [21]. XGBoost sequentially trains multiple weak models, which are then assembled using the gradient boosting method of outputs to improve prediction performance. XGBoost uses Taylor binomial expansion to approximate the objective function and arbitrary differentiable loss functions to accelerate the model construction convergence process [22]. In addition, XGBoost applies regularized boosting techniques to penalize the complexity of the model and correct overfitting, thus increasing model accuracy [21].

Finally, elastic net (EN) is a hybrid of L1 and L2 regularization, integrating the penalty terms of both. EN combines the Ridge penalty item, to achieve effective regularization, and the Lasso penalty item, to select variables, allowing for effective model learning with only a small number of arguments that are non-zero sparse, just like Lasso, but while maintaining some of Ridge’s regular properties, thus providing certain advantages as follows: 1. EN encourages group effects in the case of highly correlated variables, rather than setting some of them to 0, like Lasso. 2. Ens are useful when multiple features are correlated with one another. 3. Lasso tends to choose one of them at random, while elastic net tends to choose two [23].

**Figure 2:**
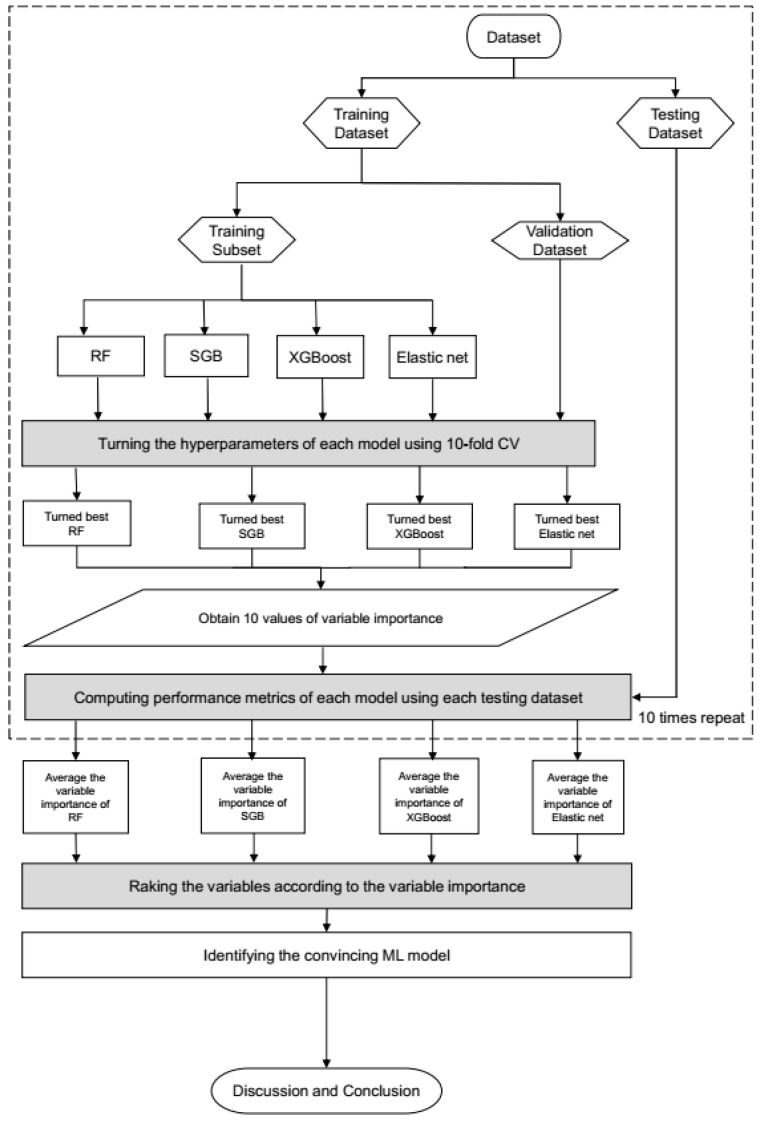
Proposed machine learning prediction scheme.

Figure 2 presents the proposed prediction and important variable identification scheme that combines the four Mach-L methods. First, patient data were collected to prepare the dataset, which was then randomly divided into a training dataset (80%) for model building and a testing dataset (20%) for model testing. In the training process, the hyperparameters of each Mach-L method must be tuned to construct an effective model. In this study, a 10-fold cross-validation technique was used for hyperparameter tuning.

The training dataset was further randomly divided into a training dataset to build the model with a different set of hyperparameters, and a validation dataset for model validation. All possible combinations of hyperparameters were investigated via grid search. The model with the lowest root mean square error on the validation dataset was taken as the best model for each Mach-L method. The best models for RF, SGB, XGBoost, and EN were generated to obtain the corresponding variable importance ranking information.

During the testing phase, the performance of the best machine learning models was evaluated using the testing dataset. Since the target variable in this study is a numerical variable, the model performance was compared using different metrics, including symmetric mean absolute percentage error (SMAPE), relative absolute error (RAE), root relative squared error (RRSE), and root mean squared error (RMSE). The values for these metrics are listed in Table 3.

To ensure a more reliable and stable comparison, the training and testing processes were each repeated 10 times. The performance metrics of the four machine learning models were then averaged for comparison against the performance of the benchmark MLR model using the same training and testing datasets. A model with an average metric lower than that of the MLR model was considered to be a more convincing model.

Because all of the machine learning methods used can rank the importance of each predictor variable, we defined the priority demonstrated in each model that was ranked 1 as the most critical risk factor, and that ranked as 25 was the last selected risk factor. The machine learning methods used in this study may produce different variable importance rankings due to their unique modeling characteristics. To maximize the stability and reliability of our findings, we integrated the variable importance rankings of the pricier machine learning models. In the final stage of our proposed scheme, we summarize and discuss our significant findings based on the pricier machine learning methods.

All methods were performed using R software version 4.0.5 and RStudio version 1.1.453, with the required packages installed [24,25].

The Materials and Methods should be described with sufficient details to allow others to replicate and build on the published results. Please note that the publication of your manuscript implicates that you must make all materials, data, computer code, and protocols associated with the publication available to readers. Please disclose at the submission stage any restrictions on the availability of materials or information. New methods and protocols should be described in detail while well-established methods can be briefly described and appropriately cited.

Research manuscripts reporting large datasets that are deposited in a publicly available database should specify where the data have been deposited and provide the relevant accession numbers. If the accession numbers have not yet been obtained at the time of submission, please state that they will be provided during review. They must be provided prior to publication.

Interventionary studies involving animals or humans, and other studies that require ethical approval, must list the authority that provided approval and the corresponding ethical approval code.

## 3. Results

**Figure 3:**
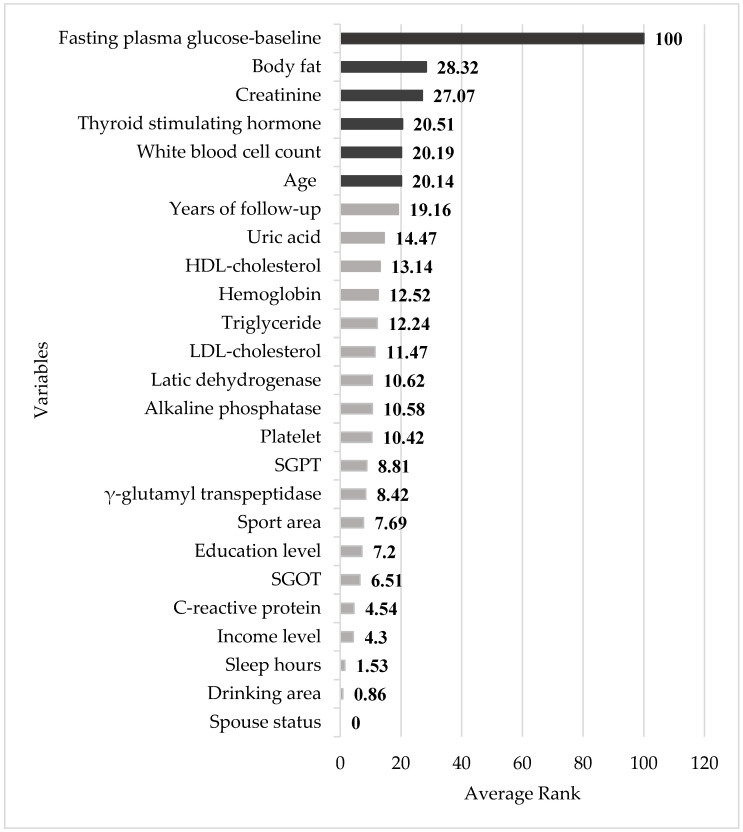
Relative importance of variables in Model 1. SGPT: Serum glutamic pyruvic transaminase, SGOT: Serum glutamic oxaloacetic transaminase.

**Figure 4:**
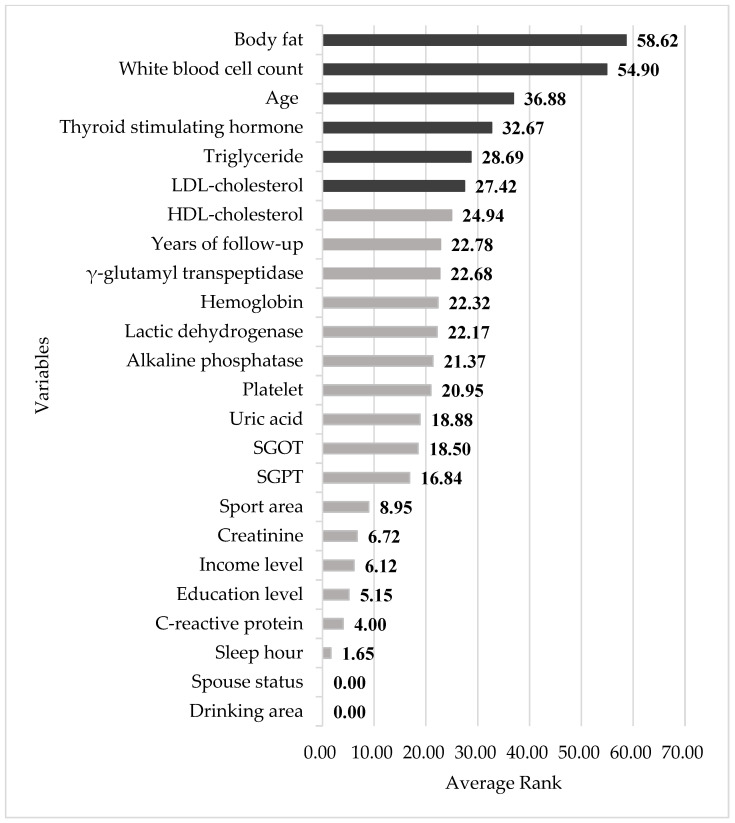
Relative variable importance in Model 2. SGPT: Serum glutamic pyruvic transaminase, SGOT: Serum glutamic oxaloacetic transaminase.

A total of 2789 study participants developed prediabetes, with age, BF, WBC, FPG_base_, γ-GT, LDH, UA, TG, and LDL-C as the most important impact factors for the total 5.8-year follow-up period, while HDL-C, TSH, and sport area also displayed significance in the earlier follow-up stages. Unmarried subjects were found to be more susceptible to developing prediabetes, while the educational level was found to have no significant impact. Subjects without income were also more susceptible (Table 1). Table 4 compares the performance of the four different methods. For both models, the four Mach-L methods produced lower values for SMAPE, RAE, RRSE, and RMSE, indicating that they outperformed MLR. Table 5 shows the importance percentage of the four Mach-L methods. The rightmost column averages the four methods, indicating that the most important factors for predicting the FPG_end_ were FPG_base_, BF, Cr, TSH, WBC, and age in Model 1. As previously noted, the importance percentage for the FPG_base_ was 100%, which is significantly higher than the second most important impact factor, i.e., BF (28.32%). Table 6 shows the results for Model 2, excluding the FPG_base_. Similar to Model 1, the most important factors are BF, WBC, age, TSH, TG, and LDL-C. Finally, Figure 3 and Figure 4, respectively, present illustrations of the results in Table 5 and Table 6, allowing for closer observations of the risk factor rankings.

**Table 4:** The average performance of linear regression and the four machine learning methods.

A. Model 1				
Methods	SMAPE	RAE	RRSE	RMSE
MLR	0.0534	0.9349	0.951	6.5015
RF	0.0535	0.9358	0.9531	6.5154
SGB	0.0533	0.9323	0.9503	6.4962
XGBoost	0.0533	0.9333	0.9535	6.5184
Elastic net	0.0534	0.935	0.9516	6.5055
**B. Model 2**				
**Methods**	**SMAPE**	**RAE**	**RRSE**	**RMSE**
MLR	0.054	0.9861	0.9873	6.4317
RF	0.0535	0.978	0.9814	6.3931
SGB	0.0538	0.983	0.9843	6.4124
XGBoost	0.0536	0.9792	0.9828	6.4027
Elastic net	0.0538	0.9832	0.9851	6.4174

MLR: multiple linear regression, RF: random forest, SGB: stochastic gradient boosting, XGBoost: eXtreme Gradient Boosting, SMAPE: symmetric mean absolute percentage error, RAE: relative absolute error, RRSE: root relative squared error, and RMSE: root mean squared error.

**Table 5:** Importance percentages of risk factors predicting future fasting plasma glucose using four different machine learning methods in Model 1.

Variables	RF	SGB	XGBoost	Elastic Net	MOIP
Age	29.79	11.12	14.7	24.96	20.14
Years of follow up	30.94	7.49	9.43	28.81	19.16
Body fat	53.75	20.11	21.23	18.19	28.32
Leukocyte	37.63	2.98	3.66	36.49	20.19
Hemoglobin	33.73	0.69	1.01	14.65	12.52
Platelet	39.43	0.86	0.89	0.52	10.42
Fasting plasma glucose—baseline	100	100	100	100	100
SGPT	30.6	3.15	0.97	0.53	8.81
SGOT	26.07	0	0	0	6.51
γ-glutamyl transpeptidase	31.78	0.57	1.35	0	8.42
Latic dehydrogenase	37.77	2.26	2.09	0.37	10.62
Uric acid	36.73	0.53	0.93	19.71	14.47
Creatinine	14.76	0	0	93.54	27.07
Triglyceride	40.42	3.76	4.81	0	12.24
HDL-cholesterol	36.64	4.15	5.12	6.68	13.14
LDL-cholesterol	38.55	3.82	2.51	1.03	11.47
Alkaline phosphatase	38.63	1.6	1.92	0.2	10.58
Thyroid stimulating hormone	41.65	1.77	2.19	36.43	20.51
C-reactive protein	7.37	0	0	10.79	4.54
Alcohol consumption	3.23	0	0	0.24	0.86
Sport area	19.91	2.77	3.62	4.47	7.69
Marital status	0	0	0	0	0
Sleep hours	4.71	0	0.64	0.77	1.53
Education level	9.51	0	0	19.31	7.20
Income level	9	1.11	0	7.11	4.30

RF: random forest, SGB: stochastic gradient boosting, XGBoost: eXtreme Gradient Boosting, SGPT: Serum glutamic pyruvic transaminase, SGOT: Serum glutamic oxaloacetic transaminase, MOIP: mean of importance percentage.

The most important sixth rank1st2nd3rd4th5th6th

**Table 6:** Importance percentages of risk factors predicting future fasting plasma glucose using four different machine learning methods in Model 2.

Variables	RF	SGB	XGBoost	Elastic Net	MOIP
Age	61.18	43.94	60.64	53.82	36.87
Years of follow up	56.58	8.35	16.01	18.83	22.78
Body fat	100	100	100	55.4	58.62
Leukocyte	78.4	19.31	36.78	100	54.89
Hemoglobin	71.74	8.62	10.35	0	22.32
Platelet	79.12	0	6.35	0	20.95
SGPT	70.84	0	3.15	0	16.83
SGOT	65.09	3.83	6.61	0	18.49
γ-glutamyl transpeptidase	69.96	10.26	10.39	0.52	22.67
Latic dehydrogenase	80.21	0	9.07	0	22.16
Uric acid	72.78	1.68	9.34	0	18.88
Creatinine	35.81	0	0	0	6.71
Triglyceride	82.98	18.88	28.27	0.53	28.69
HDL-cholesterol	78.35	9.5	12.86	8.98	24.94
LDL-cholesterol	82.46	12.76	17.25	2.3	27.42
Thyroid stimulating hormone	82.99	3.33	12.38	48.8	32.66
C-reactive protein	20.59	0	0	0	4.00
Alcohol consumption	6.6	0	0	0	0
Sport area	44.13	5.69	12.24	5.28	8.95
Marital status	0	0	0	0	0
Sleep hours	11.25	0	4.76	0	1.65
Education level	24.46	0	0	0	5.14
Income level	26.86	0	0	0	6.11

RF: random forest, SGB: stochastic gradient boosting, XGBoost: eXtreme Gradient Boosting, SGPT: Serum glutamic pyruvic transaminase, SGOT: Serum glutamic oxaloacetic transaminase, MOIP: mean of importance percentage.

The most important sixth rank1st2nd3rd4th5th6th

## 4. Discussion

The present study followed 6247 young ethnically Chinese men for an average of 5.8 years. The subject data included lifestyle information, allowing for a more comprehensive view of the predictors for glucose change. Using four different Mach-L in Model 1, we found that FPG_base_, BF, Cr, TSH, WBC, and age were the six most important factors for the FPG_end_. Given the disproportionate impact of the FPG_base_ on the second most important factor (100% versus 28.3% for BF), Model 2 was built excluding the FPG_base,_ and the same methods were repeated, finding only minor differences in terms of the key impact variables.

Consistent with other studies, the FPG_base_ was found to be the leading determinator for an increased FPG_end_. In 2021, We et al. found that FPG was the most important predictor for prediabetes in a 3.35-year follow-up period among 551 Chinese subjects, aged from 40–70 years old [26]. However, that study used multiple logistic regression and provided a hazard ratio (HR: 2.284; 95% confidence interval: 1.556, 3.352; *p* < 0.001). Logistic regression is less informative than MLR because it does not present quantitative changes of the relationships between the dependent and independent variables. Another review article published by Abdul-Ghani et al. also supported the role of FPG. They reported the development of a variety of multivariate models, all of which were useful for predicting future T2D. The main pathophysiology underlines how the FPG might be related to the decline of β-cell function with increasing age [27]. Our results further confirm that even a mild elevation of FPG might lead to the further dysregulation of glucose metabolism.

In both Models 1 and 2, BF was the second most important risk factor. While the present study accounts for BMI, BF is more accurate and was thus used to build the models [28]. As noted in the Methods section, the impact of BF was much less significant than that of FPG. To demonstrate the effects of BF on glucose metabolism, Jo et al. [29] classified 6335 participants from the National Health and Nutrition Examination Survey into four groups as follows: (1) normal weight with normal %BF, (2) normal weight with high %BF, (3) overweight with normal %BF, and (4) overweight with high % BF. The most important finding was that the prevalence of abnormal glucose in the normal weight group with a high % of BF (13.5%) is significantly higher than that of the overweight group with a low % of BF (10.5%, *p* < 0.001). This finding is incompatible with our result, which further supports the importance of BF in glucose metabolism. BF is positively related to plasma levels of free fatty acid [30], which has a significantly negative impact on glucose metabolism via an increased hepatic glucose output and decreased skeletal muscle glucose disposal, thus producing inflammatory proteins and increasing insulin resistance [31,32,33]. These effects clearly explain the present findings.

The WBC was the 5th and 2nd important factor in Model 1 and 2, respectively. There were many studies showing that this relationship does exist [34,35,36,37]. For example, Jiang et al. showed that the WBC was positively correlated with glycated hemoglobin and 2 h postprandial glucose in 9697 Chinese [38]. It is well known that one’s WBC is closely related to oxidative stress, and could even be used in clinical caring for type 2 diabetes [39,40]. Thus, this relationship is easily understood since a high WBC, which is a marker for inflammation, is related to high TG and low LDL-C and hypertension [41,42]. All these derangements are hallmarks of insulin resistance [43].

The impact of aging on glucose metabolism has been studied extensively [44]. In the present study, age is, respectively, the 6th and 3rd most important impact factor in Models 1 and 2. Chia et al. found that the incidence of several important impairments related to glucose metabolism increases with age, including confounding impacts on insulin secretion [45,46], pulsatile insulin secretion [47], reduced β-cell response to incretin [48], and even insulin resistance [49]. The results of the present study are consistent with these findings.

TSH was the 4th most important risk factor for predicting the FPG_end_ in the present study. While this relationship is less widely known, many studies have shown that both hyper- and hypothyroidism are related to T2D [50,51,52,53]. Thyroid hormone levels affect the glucose metabolism through the following mechanisms: increased glucose absorption, gluconeogenesis and glycogenolysis, and free fatty acids via promoting lipolysis [54]. All these impacts could explain our present findings.

Finally, in Model 2, higher TG and LDL-C levels were positively correlated with the FPG_end_. Insulin resistance is one of the main causes for T2D [55], while major changes to the lipid profile include increased TG and LDL-C [56]. Therefore, our results are consistent with previous findings.

It is interesting to note that the plasma Cr level was selected in Model 1, but not in Model 2. This could be explained by the interplay between the plasma Cr level and the FPG_base_. Yoshida et al. reported that a lower Cr level is associated with a higher chance of prediabetes [57]. When removing the baseline FPG, the position of Cr moved from 3rd to 18th in the present study. This indicates the importance of Cr and FPG_base_ being synchronized together.

Other hidden but important information should also be pointed out. In our study, the gap between the follow-up, income, education level, sleep hour, drinking status, and the presence of a spouse were all unimportant factors for determining the FPG_end_.

The present study is subject to certain limitations. First, none of the subjects were smokers, thus the impact of tobacco consumption cannot be determined. Secondly, the MJ Health Screening cohort generally excludes those with lower socio-economic statuses who cannot afford the company’s services, thus the sample may be subject to selection bias. Finally, our study was limited to ethnic Chinese subjects, and caution should be taken in extrapolating the findings to other ethnic groups.

## 5. Conclusions

Mach-L was found to outperform traditional MLR in terms of capturing non-linear relationships. FPG_base_, BF, WBC, age, TSH, TG, and LDL-C were the most important determinators for the FPG_end_ after 5.8 years in a group of Chinese men, aged from 18 to 35 years old.

## Figures and Tables

**Table 1:** Participant descriptive data.

Variable	Mean ± SD
n	6247
Age (year)	27.7 ± 5.1
Years of follow-up	5.8 ± 4.2
Body fat (mg/dL)	22.3 ± 5.4
Leukocyte (×10^3^/μL)	6.2 ± 1.4
Hemoglobin (×10^6^/μL)	15.4 ± 0.9
Platelets (×10^3^/μL)	236.7 ± 49.5
Fasting plasma glucose—baseline (mg/dL)	92.0 ± 4.7
Fasting plasma glucose—end of follow-up (mg/dL)	97.2 ± 6.8
Serum glutamic pyruvic transaminase (IU/L)	31.5 ± 47.7
Serum glutamic oxaloacetic transaminase (IU/L)	24.1 ± 20.8
Serum γ-glutamyl transpeptidase (IU/L)	19.8 ± 16.9
Lactate dehydrogenase (IU/L)	287.8 ± 66.7
Uric acid (mg/dL)	7.0 ± 1.4
Creatinine (mg/dL)	1.0 ± 0.1
Triglyceride (mg/dL)	100.3 ± 60.9
High density lipoprotein cholesterol (mg/dL)	49.2 ± 11.8
Low density lipoprotein cholesterol (mg/dL)	112.5 ± 31.1
Alkaline phosphatase	147.3 ± 47.3
Thyroid stimulating hormone (IU/mL)	1.6 ± 1.6
C-reactive protein (mg/dL)	0.2 ± 0.4
Drinking area	1.6 ± 7.2
Sport area	9.5 ± 9.0
Spouse status	
Single	3957 (63.9%)
With spouse	2232 (36.1%)
Sleep hours	
0–4 h/day	24 (0.4%)
4–6 h/day	1054 (16.9%)
6–8 h/day	4745 (76.1%)
>8 h/day	408 (6.6%)
Education level	
Primary school	3 (0.05%)
Junior high school	51 (0.8%)
Senior high school	1012 (16.3%)
College	1830 (29.4%)
University	2293 (36.9%)
Higher than a master’s degree	1031 (16.6%)
Income level (thousand USD/year)	
0/year	1232 (19.7%)
12.7/year	1029 (16.5%)
12.7–25.3/year	2822 (45.2%)
25.3–38.0/year	883 (14.1%)
38.0–50.6/year	130 (2.1%)
50.6–63.3/year	73 (1.2%)
>63.3/year	78 (1.2%)

**Table 2:** The results of correlation between risk factors and fasting plasma glucose at the end of the follow-up.

Variable	Value
FPG_base_	0.301 **
Body fat	0.139 **
Age	0.121 **
TG	0.095 **
LDL-C	0.087 **
WBC	0.064 **
γ-GT	0.058 **
UA	0.053 **
LDH	0.037 **
GPT	0.033 *
Drink area	0.023
Hb	0.020
Platelets	0.012
GOT	0.012
CRP	0.008
Gap year	0.006
HDL-C	−0.086 **
Sport area	−0.058 **
TSH	−0.018
ALP	−0.016
Cr	−0.001
Sleep time	−0.006

FPG_base_: fasting plasma glucose at the baseline of the follow-up, WBC: white blood cell count, Hb: hemoglobin, ALP: alkaline phosphatase, GOT: serum glutamic oxaloacetic transaminase, GPT: serum glutamic pyruvic transaminase,γ-GT: serum γ-glutamyl transpeptidase, LDH: lactate dehydrogenase, UA: uric acid, TG: triglyceride, HDL-C: high-density lipoprotein cholesterol, LDL-C: low-density lipoprotein cholesterol, TSH: thyroid-stimulating hormone, CRP: C-reactive protein, Cr: creatinine, * *p* < 0.01, ** *p* < 0.001.

**Table 3:** Four performance metrics used: stochastic gradient boosting, random forest, eXtreme gradient boosting, and elastic net.

Metrics	Description	Calculation
SMAPE	Symmetric Mean Absolute Percentage Error	$SMAPE=\frac{1}{n}\sum_{i=1}^{n}\frac{\left|y_{i}-{\hat{y}}_{i}\right|}{\left(\left|y_{i}\right|+\left|{\hat{y}}_{i}\right|\right)/2}\times 100$
RAE	Relative Absolute Error	$RAE=\sqrt{\frac{\sum_{i=1}^{n}{\left(y_{i}-{\hat{y}}_{i}\right)}^{2}}{\sum_{i=1}^{n}{\left(y_{i}\right)}^{2}}}$
RRSE	Root Relative Squared Error	$RRSE=\sqrt{\frac{\sum_{i=1}^{n}{\left(y_{i}-{\hat{y}}_{i}\right)}^{2}}{\sum_{i=1}^{n}{\left(y_{i}-\bar{Y}\right)}^{2}}}$
RMSE	Root Mean Squared Error	$RMSE=\sqrt{\frac{1}{n}\sum_{i=1}^{n}{\left(y_{i}-{\hat{y}}_{i}\right)}^{2}}$

## Data Availability

Data available on request due to privacy/ethical restrictions.

## References

[1] Zueger T., Schallmoser S., Kraus M., Saar-Tsechansky M., Feuerriegel S., Stettler C. (2022). Machine learning for predicting the risk of transition from prediabetes to diabetes. Diabetes Technol. Ther..

[2] Kushwaha S., Srivastava R., Jain R., Sagar V., Aggarwal A.K., Bhadada S.K., Khanna P. (2022). Harnessing machine learning models for non-invasive pre-diabetes screening in children and adolescents. Comput. Methods Programs Biomed..

[3] Lawrence J.M., Divers J., Isom S., Saydah S., Imperatore G., Pihoker C., Marcovina S.M., Mayer-Davis E.J., Hamman R.F., Dolan L. (2021). Trends in Prevalence of Type 1 and Type 2 Diabetes in Children and Adolescents in the US, 2001–2017. Jama.

[4] Wang C.K., Chang C.Y., Chu T.W., Liang Y.J. (2023). Using Machine Learning to Identify the Relationships between Demographic, Biochemical, and Lifestyle Parameters and Plasma Vitamin D Concentration in Healthy Premenopausal Chinese Women. Life.

[5] Zoungas S., Woodward M., Li Q., Cooper M.E., Hamet P., Harrap S., Heller S., Marre M., Patel A., Poulter N. (2014). Impact of age, age at diagnosis and duration of diabetes on the risk of macrovascular and microvascular complications and death in type 2 diabetes. Diabetologia.

[6] Choi B.C., Shi F. (2001). Risk factors for diabetes mellitus by age and sex: Results of the National Population Health Survey. Diabetologia.

[7] Peng W.K. (2021). Clustering Nuclear Magnetic Resonance: Machine learning assistive rapid two-dimensional relaxometry mapping. Eng. Rep..

[8] Veiga M.I., Peng W.K. (2020). Rapid phenotyping towards personalized malaria medicine. Malar. J..

[9] Mitchell T. (1997). Machine Learning.

[10] Nusinovici S., Tham Y.C., Yan M.Y., Ting D.S., Li J., Sabanayagam C., Wong T.Y., Cheng C.Y. (2020). Logistic regression was as good as machine learning for predicting major chronic diseases. J. Clin. Epidemiol..

[11] Wu X., Tsai S.P., Tsao C.K., Chiu M.L., Tsai M.K., Lu P.J., Lee J.H., Chen C.H., Wen C., Chang S.S. (2017). Cohort Profile: The Taiwan MJ Cohort: Half a million Chinese with repeated health surveillance data. Int. J. Epidemiol..

[12] MJ Health Research Foundation (2016). The Introduction of MJ Health Database.

[13] MJ Health Research Foundation (2014). MJ Health Survey Database, MJ BioData [Data File], MJ BioBank [Biological Specimen]. Available from MJ Health Research Foundation. http://www.mjhrf.org.

[14] Latest ADA Annual Standards of Care Includes Changes to Diabetes Screening, First-Line Therapy, Pregnancy, and Technology. https://diabetes.org/newsroom/press-releases/2021/latest-ada-annual-standards-of-care-includes-changes-to-diabetes-screening-first-line-therapy-pregnancy-technology.

[15] Wu C.Z., Huang L.Y., Chen F.Y., Kuo C.H., Yeih D.F. (2023). Using Machine Learning to Predict Abnormal Carotid Intima-Media Thickness in Type 2 Diabetes. Diagnostics.

[16] Wang M.L. (2016). MJ Health Screening Equipment Use and Replacement Records.

[17] Breiman L. (2001). Random Forests. Mach. Learn..

[18] Calle M.L., Urrea V. (2011). Letter to the editor: Stability of Random Forest importance measures. Brief Bioinform..

[19] Chen C.-H., Wang C.-K., Wang C.-Y., Chang C.-F., Chu T.-W. (2023). Roles of Biochemistry Data, Life Style and Inflammation in Identifying Abnormal Renal Function among Elderly Chinese. World J. Clin. Cases.

[20] Friedman J.H. (2001). Greedy function approximation: A gradient boosting machine. Ann. Stat..

[21] Chen T., Guestrin C. (2016). XGBoost: A Scalable Tree Boosting System. Proceedings of the 22nd ACM SIGKDD International Conference on Knowledge Discovery and Data Mining.

[22] Torlay L., Perrone-Bertolotti M., Thomas E., Baciu M. (2017). Machine learning-XGBoost analysis of language networks to classify patients with epilepsy. Brain Inform..

[23] Tay J.K., Narasimhan B., Hastie T. (2023). Elastic Net Regularization Paths for All Generalized Linear Models. J. Stat. Softw..

[24] Tool R. (2015). R Project. http://www.r-project.org/.

[25] RStudio Posit. https://posit.co/products/open-source/rstudio/.

[26] Wu J., Zhou J., Yin X., Chen Y., Lin X., Xu Z., Li H. (2021). A Prediction Model for Prediabetes Risk in Middle-Aged and Elderly Populations: A Prospective Cohort Study in China. Int. J. Endocrinol..

[27] Chiu T.H., Huang H.Y., Chiu Y.F., Pan W.H., Kao H.Y., Chiu J.P., Lin M.N., Lin C.L. (2014). Taiwanese vegetarians and omnivores: Dietary composition, prevalence of diabetes and IFG. PLoS ONE.

[28] Ranasinghe C., Gamage P., Katulanda P., Andraweera N., Thilakarathne S., Tharanga P. (2013). Relationship between Body Mass Index (BMI) and body fat percentage, estimated by bioelectrical impedance, in a group of Sri Lankan adults: A cross sectional study. BMC Public Health.

[29] Jo A., Mainous A.G. (2018). Informational value of percent body fat with body mass index for the risk of abnormal blood glucose: A nationally representative cross-sectional study. BMJ Open.

[30] Mittendorfer B., Magkos F., Fabbrini E., Mohammed B.S., Klein S. (2009). Relationship between body fat mass and free fatty acid kinetics in men and women. Obesity.

[31] Boden G. (2006). Fatty acid-induced inflammation and insulin resistance in skeletal muscle and liver. Curr. Diab Rep..

[32] Boden G., Chen X., Ruiz J., White J.V., Rossetti L. (1994). Mechanisms of fatty acid-induced inhibition of glucose uptake. J. Clin. Investig..

[33] Shoelson S.E., Lee J., Yuan M. (2003). Inflammation and the IKK beta/I kappa B/NF-kappa B axis in obesity- and diet-induced insulin resistance. Int. J. Obes. Relat. Metab. Disord..

[34] Fritsche A., Häring H., Stumvoll M. (2004). White blood cell count as a predictor of glucose tolerance and insulin sensitivity. The role of inflammation in the pathogenesis of type 2 diabetes mellitus. Dtsch. Med. Wochenschr..

[35] Vozarova B., Weyer C., Lindsay R.S., Pratley R.E., Bogardus C., Tataranni P.A. (2002). High white blood cell count is associated with a worsening of insulin sensitivity and predicts the development of type 2 diabetes. Diabetes.

[36] Gokulakrishnan K., Deepa R., Sampathkumar R., Balasubramanyam M., Mohan V. (2009). Association of leukocyte count with varying degrees of glucose intolerance in Asian Indians: The Chennai Urban Rural Epidemiology Study (CURES-26). Metab. Syndr. Relat. Disord..

[37] Nakanishi N., Yoshida H., Matsuo Y., Suzuki K., Tatara K. (2002). White blood-cell count and the risk of impaired fasting glucose or Type II diabetes in middle-aged Japanese men. Diabetologia.

[38] Jiang H., Yan W.H., Li C.J., Wang A.P., Dou J.T., Mu Y.M. (2014). Elevated white blood cell count is associated with higher risk of glucose metabolism disorders in middle-aged and elderly Chinese people. Int. J. Environ. Res. Public Health.

[39] Kotani K., Sakane N. (2012). White blood cells, neutrophils, and reactive oxygen metabolites among asymptomatic subjects. Int. J. Prev. Med..

[40] Peng W.K., Chen L., Boehm B.O., Han J., Loh T.P. (2020). Molecular phenotyping of oxidative stress in diabetes mellitus with point-of-care NMR system. npj Aging Mech. Dis..

[41] Huang Z.S., Chien K.L., Yang C.Y., Tsai K.S., Wang C.H. (2001). Peripheral differential leukocyte counts in humans vary with hyperlipidemia, smoking, and body mass index. Lipids.

[42] Boucher A.A., Edeoga C., Ebenibo S., Wan J., Dagogo-Jack S. (2012). Leukocyte count and cardiometabolic risk among healthy participants with parental type 2 diabetes: The Pathobiology of Prediabetes in a Biracial Cohort study. Ethn. Dis..

[43] Singh B., Saxena A. (2010). Surrogate markers of insulin resistance: A review. World J. Diabetes.

[44] Chia C.W., Egan J.M., Ferrucci L. (2018). Age-Related Changes in Glucose Metabolism, Hyperglycemia, and Cardiovascular Risk. Circ. Res..

[45] Andres R. (1971). Aging and diabetes. Med. Clin. N. Am..

[46] Davidson M.B. (1979). The effect of aging on carbohydrate metabolism: A review of the English literature and a practical approach to the diagnosis of diabetes mellitus in the elderly. Metabolism.

[47] Meneilly G.S., Veldhuis J.D., Elahi D. (1999). Disruption of the pulsatile and entropic modes of insulin release during an unvarying glucose stimulus in elderly individuals. J. Clin. Endocrinol. Metab..

[48] Meneilly G.S., Ryan A.S., Minaker K.L., Elahi D. (1998). The effect of age and glycemic level on the response of the beta-cell to glucose-dependent insulinotropic polypeptide and peripheral tissue sensitivity to endogenously released insulin. J. Clin. Endocrinol. Metab..

[49] (2008). Prevalence of Overweight, Obesity and Extreme Obesity among Adults: United States, Trends 1976–1980 through 2005–2006.

[50] Hollowell J.G., Staehling N.W., Flanders W.D., Hannon W.H., Gunter E.W., Spencer C.A., Braverman L.E. (2002). Serum TSH, T(4), and thyroid antibodies in the United States population (1988 to 1994): National Health and Nutrition Examination Survey (NHANES III). J. Clin. Endocrinol. Metab..

[51] Perros P., McCrimmon R.J., Shaw G., Frier B.M. (1995). Frequency of thyroid dysfunction in diabetic patients: Value of annual screening. Diabet. Med..

[52] Tamez-Pérez H.E., Martínez E., Quintanilla-Flores D.L., Tamez-Peña A.L., Gutiérrez-Hermosillo H., Díaz de León-González E. (2012). The rate of primary hypothyroidism in diabetic patients is greater than in the non-diabetic population. An. Obs. Study. Med. Clin..

[53] Distiller L.A., Polakow E.S., Joffe B.I. (2014). Type 2 diabetes mellitus and hypothyroidism: The possible influence of metformin therapy. Diabet. Med..

[54] Nishi M. (2018). Diabetes mellitus and thyroid diseases. Diabetol. Int..

[55] Savage D.B., Petersen K.F., Shulman G.I. (2005). Mechanisms of insulin resistance in humans and possible links with inflammation. Hypertension.

[56] Cohn G., Valdes G., Capuzzi D.M. (2001). Pathophysiology and treatment of the dyslipidemia of insulin resistance. Curr. Cardiol. Rep..

[57] Yoshida N., Miyake T., Yamamoto S., Furukawa S., Senba H., Kanzaki S., Koizumi M., Ishihara T., Yoshida O., Hirooka M. (2019). The Serum Creatinine Level Might Be Associated with the Onset of Impaired Fasting Glucose: A Community-based Longitudinal Cohort Health Checkup Study. Intern. Med..

